# On-top plasty for late-presenting atypical Wassel type VII thumb duplication: a case report

**DOI:** 10.1097/RC9.0000000000000942

**Published:** 2026-10-06

**Authors:** Erfan Nasrullah, Heri Suroto

**Affiliations:** a Department of Orthopedics and Traumatology, R.T. Notopuro General Hospital, Sidoarjo, Indonesia; b Department of Orthopedics and Traumatology, Faculty of Medicine, Universitas Airlangga, Surabaya, Indonesia; c Department of Orthopedics and Traumatology, Dr. Soetomo General Academics Hospital, Surabaya, Indonesia; d Cell and Tissue Bank-Regenerative Medicine Center, Dr. Soetomo General Academic Hospital, Surabaya, Indonesia

**Keywords:** case report, late presentation, on-top plasty, polydactyly, thumb duplication, Wassel classification

## Abstract

**Background:**

Thumb duplication beyond early childhood presents unique reconstructive challenges. On-top plasty offers an alternative when standard excision may yield suboptimal results.

**Case presentation:**

We report a 10-year-old girl with late-presenting bilateral thumb duplication. The right hand demonstrated atypical Wassel type VII configuration with a floating ulnar thumb and hypoplastic radial triphalangeal thumb. On-top plasty was performed, combining the distal ulnar segment with the proximal radial segment. At 6-month follow-up, radiographic union was achieved with good functional outcomes including pinch and grasp activities.

**Clinical discussion:**

Wassel type VII thumb duplication presents significant reconstructive challenges, especially when anatomical dominance is indistinct. Although early intervention is recommended, late presentation necessitates strategies that account for established skeletal maturation and functional adaptation. Simple excision was considered insufficient for achieving optimal stability and esthetics. Therefore, on-top plasty was performed, combining the most robust distal and proximal components. A proximal phalanx-level osteotomy enabled preservation of native thenar insertions and collateral ligament stability, which are essential for stable opposition and pinch mechanics. This case demonstrates that individualized, anatomy-based reconstruction using on-top plasty can overcome the limitations of delayed presentation, yielding satisfactory outcomes despite advanced age at presentation.

**Conclusion:**

On-top plasty represents a viable reconstructive option for complex thumb polydactyly in late-presenting cases when neither duplicated digit offers adequate functional potential.

## Introduction

Thumb duplication, also known as preaxial polydactyly, is one of the most common congenital hand anomalies[1]. Surgical correction is generally recommended between 1 and 3 years of age to optimize functional development and cosmetic outcomes[2]. Consequently, late presentation remains uncommon. Management is often challenging because skeletal growth, soft-tissue adaptation, and functional patterns may influence surgical planning.

HIGHLIGHTS

- Late-presenting Wassel type VII thumb duplication is a rare clinical finding with limited documentation in the literature.
- Standard excision and conventional reconstruction may not provide optimal functional and esthetic outcomes in such cases.
- The application of on-top plasty in an older child represents a rarely reported surgical strategy for this subtype.
- Management emphasizes achieving esthetic symmetry and functional stability in an older child with established thumb function.

Complex duplications, particularly Wassel type VII anomalies, may not be amenable to simple excision when neither duplicated thumb alone provides satisfactory function or appearance. In such situations, on-top plasty allows reconstruction by combining the most favorable proximal and distal components of each duplicated digit.

We report a late-presenting atypical Wassel type VII thumb duplication in a 10-year-old girl with a floating ulnar thumb and a hypoplastic triphalangeal radial thumb. Because standard excision was predicted to result in suboptimal outcomes, reconstruction was performed using an on-top plasty technique. This report was prepared according to the SCARE criteria[3].

## Case presentation

A 10-year-old right-handed girl, the third of three children of non-consanguineous parents, presented with thumb duplication. She was delivered at term by cesarean section because of a transverse lie. Growth and developmental milestones were normal. There was no family history of congenital hand anomalies.

The patient had bilateral thumb duplication since birth. The left thumb duplication was classified as Wassel type IV and had been treated with standard excision and reconstruction approximately 1 year earlier, resulting in a satisfactory functional outcome.

In contrast, the right thumb demonstrated a more complex deformity. The ulnar-sided thumb showed adequate size and a well-developed nail, whereas the radial-sided thumb appeared hypoplastic. Passive motion was full, without contracture. Active motion was primarily generated at the metacarpophalangeal (MCP) and carpometacarpal (CMC) joints. No active interphalangeal (IP) joint motion was observed preoperatively in either duplicated thumb. The first web space was adequately developed and did not require lengthening (Fig. 1A). Sensation and vascularity were preserved in both duplicated digits. The patient had adapted to daily activities despite the thumb deformity. Her primary concern was cosmetic rather than functional impairment.

**Figure 1.**
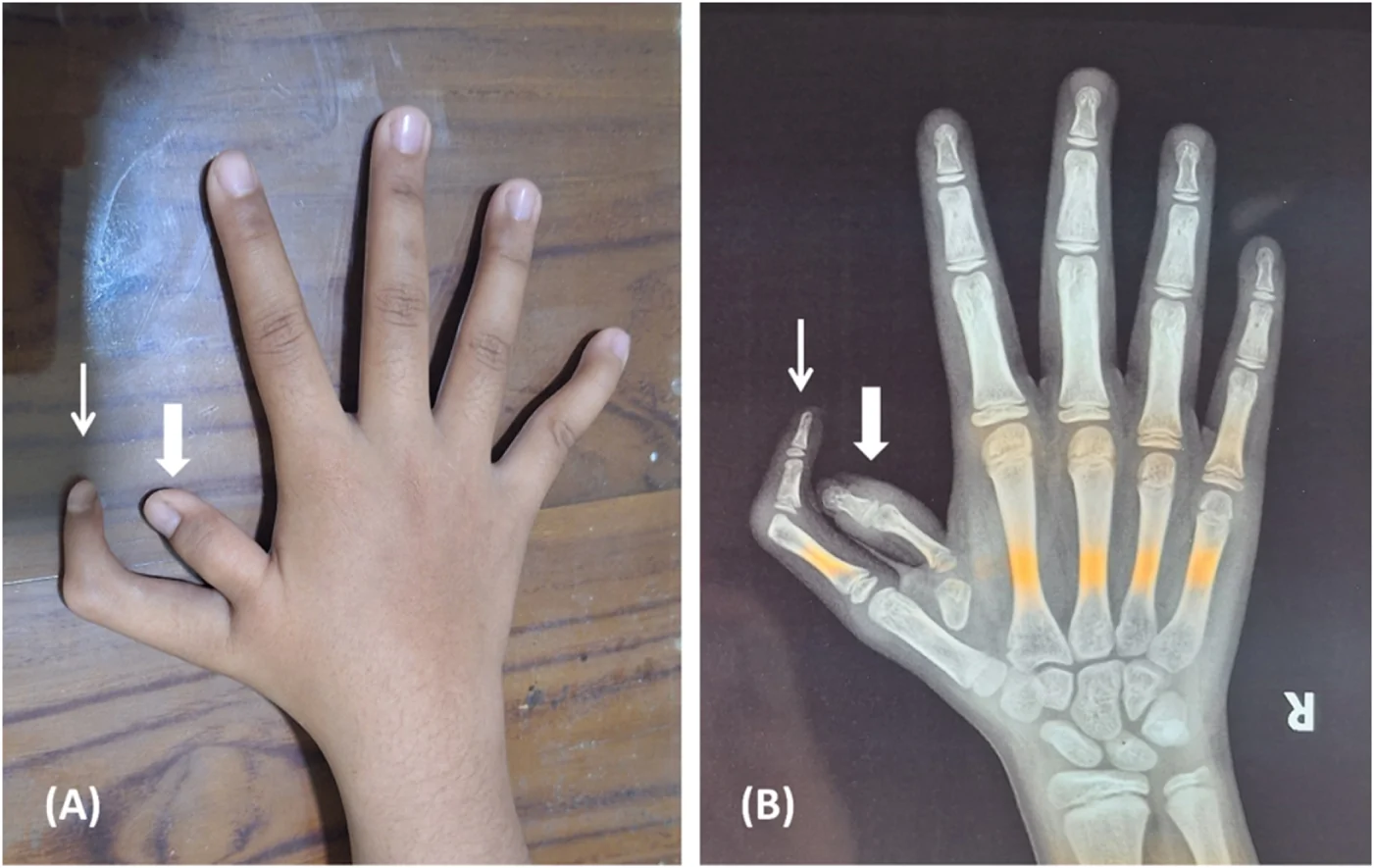
Preoperative evaluation. (A) Clinical appearance. (B) Radiographic evaluation. Thin arrows indicate a triphalangeal thumb; thick arrows indicate a floating thumb.

Radiographs demonstrated thumb duplication arising from a split metacarpal head. The ulnar component appeared as a floating digit, while the radial component demonstrated a hypoplastic triphalangeal configuration. No additional skeletal abnormalities were identified (Fig. 1B).

## Surgical technique

The procedure was performed under general anesthesia, with tourniquet control. A racket-shaped incision was made on the radial side of the duplicated thumb (Fig. 2A). Following the incision, a dorsal soft-tissue flap was developed to allow exposure of the ulnar-duplicated thumb. Dissection proceeded through the dorsal subcutaneous plane and then continued subperiosteally to expose the proximal phalanx. The duplicated extensor pollicis longus (EPL) and flexor pollicis longus (FPL) tendons of the ulnar-sided thumb were identified and preserved. Neurovascular structures were not formally isolated because this anatomical plane generally lies away from the neurovascular pedicle. Careful dissection was maintained throughout the procedure to prevent inadvertent neurovascular injury.

**Figure 2.**
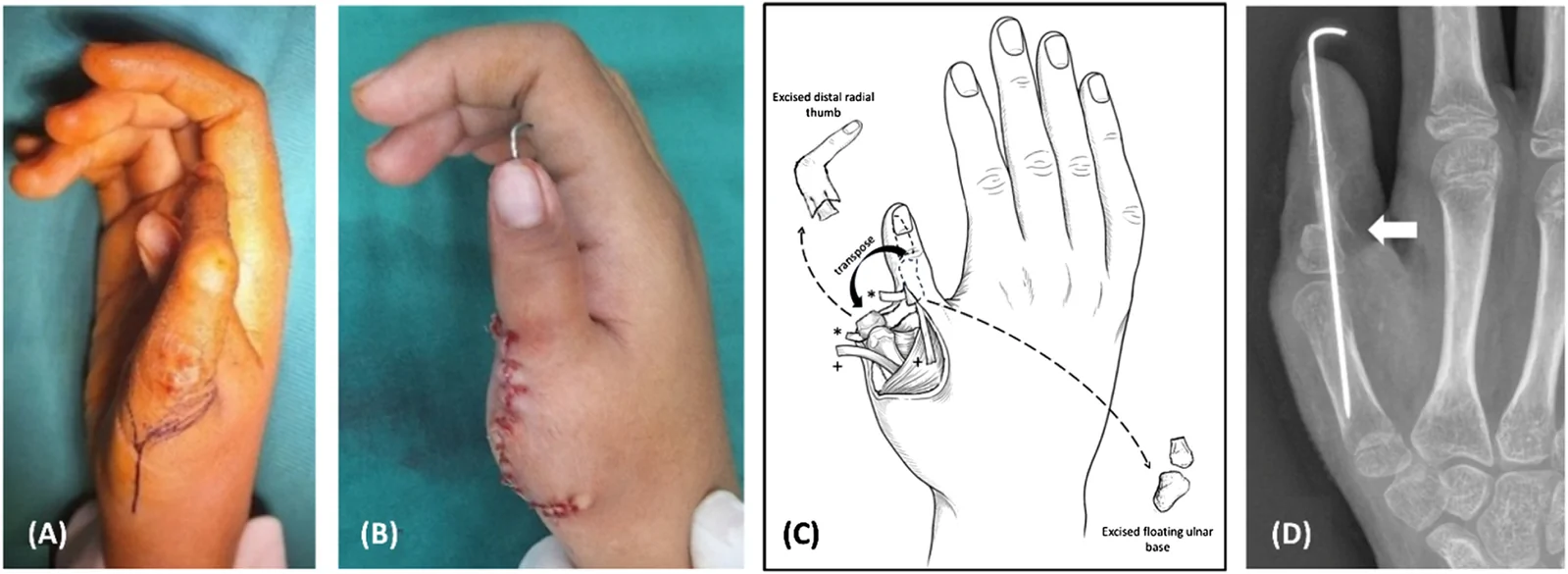
Surgical procedure and early postoperative outcomes. (A) Incision design. (B) Early postoperative clinical results. (C) Operative scheme. (^+^) EPL tendon; (*) FPL tendon. (D) Early postoperative radiograph. The arrow indicates the osteotomy site.

An osteotomy of the ulnar side of the thumb was performed at the mid-level of the proximal phalanx using a micro-sagittal saw. The proximal segment was excised, preserving the distal segment together with the nail complex and surrounding soft-tissue envelope.

The tourniquet was subsequently deflated, and distal perfusion of the ulnar thumb segment was assessed. Adequate perfusion was confirmed, and the procedure was continued on the radial side. The radially duplicated thumb was exposed, with preservation of the EPL and FPL tendons. A corresponding osteotomy was performed at the mid-level of the proximal phalanx, distal to the insertion of the thenar musculature, to preserve the established MCP joint function, followed by excision of the distal segment.

The distal segment of the ulnar-sided thumb was then transferred onto the proximal radial-sided thumb base. Length adjustment was achieved by trimming the proximal phalanx to obtain appropriate thumb length and adequate soft-tissue tension, without compromising the neurovascular structures. Rotational alignment was verified based on nail orientation. Internal fixation was performed using a 1.0-mm Kirschner wire inserted in a retrograde fashion through the distal segment and then advanced antegrade across the osteotomy site into the metacarpal.

Tendon reconstruction was subsequently performed. The EPL and FPL tendons from both duplicated thumbs were repaired end-to-end with appropriate tension. Despite the absence of preoperative active IP joint motion, tendon repair was performed to maintain continuity and a balanced thumb posture.

Redundant skin at the radial base of the thumb was excised to improve contour. Soft-tissue closure was performed in layers. Postoperatively, a thumb spica splint was applied with the thumb positioned in a functional grasp posture. Vascular viability of the transferred thumb was monitored by serial assessments of thumb color and capillary refill.

## Outcome and follow-up

Postoperative recovery was uneventful. The Kirschner wire was removed 6 weeks after surgery, followed by supervised range-of-motion exercises.

Radiographs at 3 months demonstrated satisfactory bone union. At 6 months, the reconstructed thumb maintained good alignment and appearance. Functional pinch and grasp were achieved (Fig. 3). Active IP motion remained absent, similar to the preoperative condition. Nevertheless, MCP and CMC joint function remained stable, providing satisfactory thumb function. Thumb opposition reached a Kapandji score of 8/10. Two-point discrimination was less than 6 mm at the pulp. According to the Tada scoring system, the outcome was graded as good, with a score of 4/5. The patient and family reported high satisfaction with the outcome.

**Figure 3.**
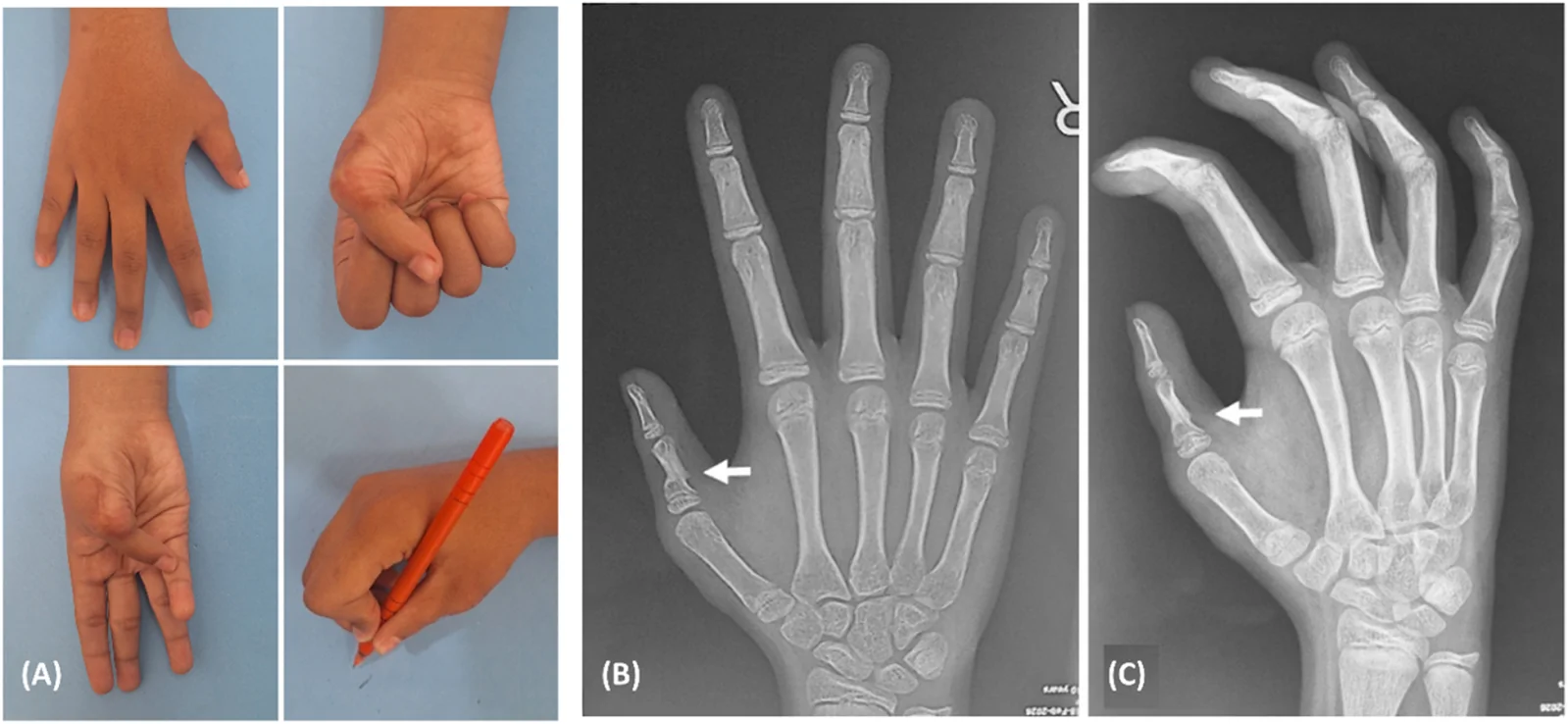
Six-month postoperative follow-up. (A) Clinical appearance and function. (B) AP radiograph. (C) Oblique radiograph. The arrow indicates the osteotomy site.

A chronological timeline summarizing the patient’s presentation, surgical intervention, and postoperative follow-up is presented in Supplemental Digital Content Figure S1, available at: https://links.lww.com/IJSCR/A153.

## Discussion

The management of thumb duplication depends on skeletal configuration, soft tissue balance, and the functional potential of each duplicated digit^[^4,5^]^. While simple excision and reconstruction are adequate in many cases, complex anomalies such as Wassel type VII may require alternative reconstructive strategies when neither duplicated thumb demonstrates a clearly dominant anatomy.

On-top plasty provides a reconstructive strategy by combining the most favorable distal and proximal components of each duplicated digit. In the present case, the ulnar-sided duplicated thumb offered superior distal soft tissue quality and nail characteristics, whereas the radial-sided thumb provided a more suitable proximal skeletal base. Neither digit alone was sufficient for reconstruction, thereby supporting the rationale for an on-top plasty approach.

On-top plasty was first described by Soiland[6] for post-traumatic digital reconstruction and was later adapted for thumb polydactyly, with several subsequent modifications reported for complex duplications[7]. Most published cases involve patients treated during early childhood. Xu *et al*[2] reported favorable outcomes following on-top plasty in children aged 11–47 months. In contrast, our patient presented at a later age (10 years).

Late presentation poses additional challenges because older children have already developed compensatory hand function, altered pinch and grasp mechanics, and psychosocial adaptation[8]. Prolonged growth may also result in progressive anatomical divergence, joint deformity, and soft tissue imbalance, requiring individualized reconstruction^[^8,9^]^.

Active IP joint motion remained absent postoperatively, possibly related to underdeveloped EPL and FPL tendon function. The lack of active IP joint motion may compromise fine pinch precision and dexterity, representing an important functional limitation. However, preserved MCP and CMC joint function allowed acceptable pinch and grasp activities, consistent with previous reports demonstrating that acceptable overall thumb function may still be achieved despite limited IP joint motion after on-top plasty reconstruction[10].

Previous authors have described metacarpal-level on-top plasty techniques for thumb duplications^[^11,12^]^. In contrast, we performed osteotomy at the proximal phalanx level, similar to Bell *et al*[13], preserving the native MCP joint and thenar musculature. Preservation of the native MCP joint and thenar musculature is an important determinant of successful thumb polydactyly reconstruction[14]. Transfer or reconstruction of thenar muscles in late-presenting cases may be technically more demanding and potentially associated with diminished functional outcomes^[^15,16^]^.

Our approach also differed from that described by Pons-Riverola *et al*[17], who emphasized neurovascular pedicle dissection during transposition. In the present case, the first web space was sufficiently wide, allowing transposition of the ulnar thumb without requiring a formal neurovascular pedicle dissection. Soft-tissue tension was adjusted through a controlled shortening osteotomy of the proximal phalanx, achieving a balance between safe soft-tissue tension and acceptable cosmetic appearance. This modification simplified the procedure while maintaining satisfactory distal perfusion^[^18,19^]^.

The presence of bilateral asymmetric thumb duplication in this patient further emphasizes the importance of anatomy-based surgical planning. While the contralateral Wassel type IV duplication was successfully managed with standard reconstruction, the more complex anatomy of the affected side required on-top plasty.

At 6 months, the patient demonstrated good alignment, effective opposition, and functional pinch and grasp. These findings suggest that on-top plasty may restore meaningful hand function in carefully selected older children. Longer-term follow-up remains necessary to assess growth-related deformity, angular deviation, joint stability, tendon balance, and patient satisfaction through skeletal maturity.

Although this report represents a single case with limited follow-up, it contributes to the existing literature by illustrating the feasibility of on-top plasty in a late-presenting atypical Wassel type VII thumb duplication.

## Conclusion

On-top plasty may be considered a reconstructive option for carefully selected cases of late-presenting complex thumb polydactyly. This case highlights the importance of individualized anatomy-based reconstruction.

## Data Availability

All data relevant to the case are included in the article.
